# Comparative transcriptomics of wild and commercial *Citrus* during early ripening reveals how domestication shaped fruit gene expression

**DOI:** 10.1186/s12870-022-03509-9

**Published:** 2022-03-17

**Authors:** Carles Borredá, Estela Perez-Roman, Manuel Talon, Javier Terol

**Affiliations:** Centro de Genómica, Instituto Valenciano de Investigaciones Agrarias (IVIA), 46113 Moncada, Valencia Spain

**Keywords:** Citrus, Domestication, RNA-seq, Allele specific expression, Fruit ripening

## Abstract

**Background:**

Interspecific hybridizations and admixtures were key in *Citrus* domestication, but very little is known about their impact at the transcriptomic level. To determine the effects of genome introgressions on gene expression, the transcriptomes of the pulp and flavedo of three pure species (citron, pure mandarin and pummelo) and four derived domesticated genetic admixtures (sour orange, sweet orange, lemon and domesticated mandarin) have been analyzed at color break.

**Results:**

Many genes involved in relevant physiological processes for domestication, such sugar/acid metabolism and carotenoid/flavonoid synthesis, were differentially expressed among samples. In the low-sugar, highly acidic species lemon and citron, many genes involved in sugar metabolism, the TCA cycle and GABA shunt displayed a reduced expression, while the P-type ATPase *CitPH5* and most subunits of the vacuolar ATPase were overexpressed. The red-colored species and admixtures were generally characterized by the overexpression in the flavedo of specific pivotal genes involved in the carotenoid biosynthesis, including *phytoene synthase*, *ζ-carotene desaturase*, *β-lycopene cyclase* and *CCD4b*, a carotenoid cleavage dioxygenase. The expression patterns of many genes involved in flavonoid modifications, especially the flavonoid and phenylpropanoid O-methyltransferases showed extreme diversity. However, the most noticeable differential expression was shown by a chalcone synthase gene, which catalyzes a key step in the biosynthesis of flavonoids. This *chalcone synthase* was exclusively expressed in mandarins and their admixed species, which only expressed the mandarin allele. In addition, comparisons between wild and domesticated mandarins revealed that the major differences between their transcriptomes concentrate in the admixed regions.

**Conclusion:**

In this work we present a first study providing broad evidence that the genome introgressions that took place during citrus domestication largely shaped gene expression in their fruits.

**Supplementary Information:**

The online version contains supplementary material available at 10.1186/s12870-022-03509-9.

## Background

Citrus are among the major fruit crops worldwide, with oranges, grapefruits, lemons and mandarins as the most economically relevant cultivars. Most of these commercial citrus are not pure species but genetic admixtures derived from interspecific hybrids that display genomic fragments from wild species, which are pure and hence free of introgressions, including citron, pummelo and mandarin [1]. Previous studies have classified mandarins based on the proportion of their genome containing introgressions: pure mandarins, which are generally considered wild species, were classified as type 1 mandarins by Wu et al. [1], and are not edible and small in size. In contrast, commercial mandarins are largely appreciated for their palatability, which has been associated with several pummelo introgressions. These admixed mandarins are classified as type 2 or type 3 mandarins, depending on whether the proportion of the genome including a pummelo haplotype is lower or higher than 10% [1, 2]. Other well-known citrus cultivars such as sweet oranges and grapefruits are also mandarin/pummelo admixtures, although they show larger and more frequent pummelo introgressions, with some genomic regions displaying two pummelo alleles [3]. As direct hybrids of mandarin x pummelo cross, sour oranges display two complete parental haplotypes. Lemons, that resulted from of a cross of sour orange and citron [4], have one complete citron haplotype, while the other one shows the admixture produced by the mandarin and pummelo ancestries [4, 5].

Genomic analyses suggest that the specific admixture patterns of each citrus cultivar largely determines its phenotype, and might imply human participation [1, 3, 6]. The generation of such admixtures is a complex process that requires crosses between pure species followed by backcrosses and/or crosses with other admixtures. This process has been related to the domestication of citrus species, together with the selection and propagation of the admixed individuals showing desirable traits. The complex relatedness network shared by mandarins, oranges and grapefruits, suggesting that they all share some recent common ancestors, supports this proposal [1].

Human-mediated selection has been the major driving force behind crop domestication, a process that is still ongoing nowadays. This selection unknowingly targeted structural and regulatory genes, producing effects that propagate through the whole transcriptome [7]. For this reason, domestication has profound effects in gene expression, affecting specific genes associated with the agronomical traits targeted by the domestication process but often altering the transcriptome at a global level too. This way, comparative transcriptomic revealed patterns of selection in domesticated tomato [8, 9], and RNA-seq performed at the population level showed how artificial selection greatly shaped the tomato transcriptome, altering the fruit sugar content and resistance to abiotic and biotic stresses [10]. Reshaping of the maize transcriptome by domestication has been also analyzed by expression profiling analyses, identifying several genes that may have contributed to this process [11].

Knowledge of the genetic changes that occurred during the domestication and breeding of perennial trees is limited, although RNA sequencing analysis of wild, landrace, and improved cultivars of pear (*Pyrus pyrifolia*), for instance, revealed specific patterns of domestication and improvement, many of them highly associated with important fruit traits [12]. Evolutionary transcriptomics has been also used to reveal the origins of olive trees (*Olea europaea*) and the genomic changes associated with their domestication, showing how the domestication of this species had a moderate impact at genome level and that the domestication syndrome was mainly related to changes in gene expression, consistent with the olive tree evolutionary history [13].

Citrus fruits display a wide variability in size, shape, color and flavor, which make them attractive to the markets. Despite this broad phenotypic diversity, genomic studies have reported a highly conserved genome, both in structure and gene content. Thus, all the *Citrus* analyzed genomes are organized in 9 chromosomes, that show an almost perfect synteny [4, 14], as well as a very similar number of highly conserved genes [15, 16]. Therefore, the variability found in citrus must rely in other factors, and changes at the expression level might appear as some of the influential ones that could be ultimately associated to the domestication of *Citrus* species.

As fruit quality is a direct consequence of the ripening process, much effort has been made to analyze maturation at different levels, and recently several studies have used transcriptomic approaches to unveil the genetic mechanisms controlling citrus fruit ripening. These works focused on the study of the regulation of commercially relevant traits such as fruit taste [17], peel color [18] and health-promoting properties [19].

Taste is largely determined by the acidity to sweetness balance, which sets towards the end of the ripening process. Acidity mostly depends on pH and titratable acid concentration [20, 21], while sweetness relies on total sugar concentration, that is simply determined measuring the total soluble solid content (TSS). While TSS has been directly linked to fruit taste in many commercial fruits, extreme juice acidity might mask sugar content and dominate flavor perception [22]. In some acidic citrus, the vacuolar lumen in the cells of the pulp vesicles reaches pH values as low as 2, more than five points below the cytoplasmic one [23]. This steep pH gradient is promoted by the citrate vacuolar intake [24], which buffers the vacuolar lumen and allows a continuous proton intake that maintains the low vacuolar pH [25]. Despite its central role in this process, citrate biosynthesis does not appear to be directly correlated with its accumulation in citrus pulp [17, 26], which mostly depends on its degradation [27, 28] and storage in the cell vacuoles [22, 29]. In non-acidic commercial citrus such as sweet oranges and clementines, sugar accumulation is a major trait for fruit quality. This accumulation is the result of a metabolic change from sucrose utilization to storage [30], which increases sucrose concentration in the pulp [31]. As fruit ripens, expression levels of sugar invertases (*INV*) drop, while sucrose phosphate synthases (*SPS*), sucrose phosphatases (*SPP*) and sucrose synthases (*SuSy*), all involved in sucrose synthesis, increase their expression [32], all of which result in the accumulation of sucrose and its derivatives in the pulp during the late ripening stages [33].

Color is another major agronomical trait of citrus fruits, mostly produced by the accumulation of carotenoids. During color break, peel chlorophylls are hydrolyzed [34] while carotenoid biosynthesis is promoted in the chromoplasts [35] and, in red-colored fruits, the carbon flux is redirected towards the production of β-carotene and its derivatives [36]. The differential accumulation profile of carotenoids and apocarotenoids generates the broad range of colors observed in *Citrus* species, a subject thoroughly studied given the commercial relevance of their fruits [reviewed in [37]. Specifically, the bright red color found in many citrus is produced by the accumulation of C30 apocarotenoids such as β-citraurin, while other carotenoids such as violaxanthin also contribute to the final color [38]. In contrast, yellow and non-colored fruits such as pummelos, citrons and lemons display lower carotenoid contents [39–41]. In the pulp vesicles of most citrus species, the accumulation of carotenoid derivatives follows a similar process, although it starts earlier and reaches lower values overall [42, 43].

Citrus well-known health benefits [44] result from the large number of bioactive compounds, like flavonoids, found in their fruits. Recent studies have highlighted the vast flavonoid diversity existing in *Citrus* species, especially that of polymethoxylated flavonoids and O-glycosylated flavonoids on the fruit flavedo, where their concentration is higher [19]. Moreover, flavonoid profiles in different citrus are extremely variable between species and admixtures, even allowing their clustering based on such profiles that greatly resembles the phylogenetic tree of the genus *Citrus* [45]. This variability might be the result of the expansion of the genic family of flavonoid O-methyltransferases that characterize citrus genomes [46].

In this work, we have used the RNA-Seq technology to address how domestication shaped gene expression in pure *Citrus* species, analyzing transcriptomic changes caused by hybridization and admixtures, to generate the attractive commercial varieties we enjoy nowadays. While fruit ripening has been thoroughly studied in citrus, most works have focused on comparative studies of somatic mutants or closely related cultivars [18, 47, 48], but no genus-wide analysis has been performed so far. We have performed a comprehensive transcriptomic analysis of flavedo and pulp of fruits of seven different citrus cultivars at the time of color break. Three of them are pure species and belong to the main citrus taxonomic groups (citrons, pummelos and mandarins), while the remaining four are commercial varieties with varying admixture levels. Using a novel approach that involves the analysis of genomic unbalance and allele-specific expression, we provide new insights of the effects of citrus hybridizations and domestication on gene expression during ripening.

## Results

### Physiological characterization of ripening fruits

**Fig. 1:**
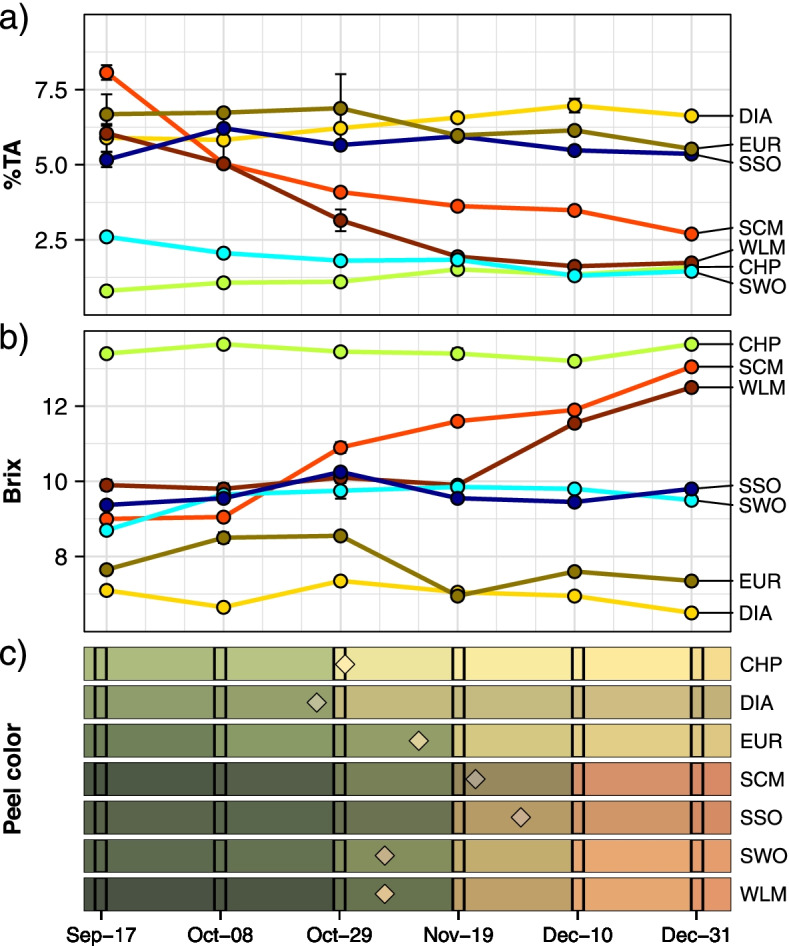
Phenotyping of the selected accessions across three months in six timepoints. **a** Average titratable acid content per sample across three replicates. Vertical bars represent the standard error for each measurement. **b** Average Brix degrees per sample. Vertical bars represent the standard error for each measurement. **c** Average color per sample at six measuring times, indicated by the sampling date. Color was calculated from the L*a*b values provided by the Minolta colorimeter. The color values between measurements were interpolated. Diamonds mark the color and date at which samples were collected and processed for RNA-seq sequencing. CHP: *C. maxima*, DIA: *C. medica*, EUR: *C. limon*, SCM: *C. reticulata*, SSO: *C. aurantium*, SWO: *C. sinensis*, WLM: *C. deliciosa*

Fruit acidity and sugar content of the seven selected species were measured during the ripening process. Major changes in juice acidity were only observed in the two mandarins, whose acidity decreased considerably as ripening progressed (Fig. 1). Citron, lemon and sour orange were characterized by a titratable acid (TA) content above 5% during the whole period; conversely, sweet orange and pummelo showed a constant, low level of TA content (Fig. 1a). Sugar content (measured in Brix degrees, or °Brix) increased considerably in the two mandarins, while it remained invariably high in pummelo, very low in lemon and citron, and in intermediate values in the two oranges (Fig. 1b). The color break and the fruit color at the time of sampling is shown in Fig. 1c.

### RNA-seq read mapping and sample clustering

**Fig. 2:**
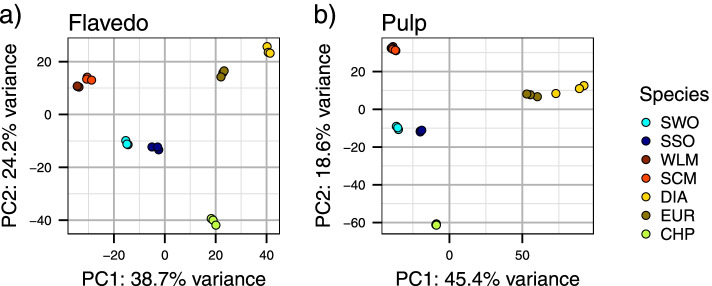
Principal component analysis of expression data. Each replicate is shown as an independent dot; flavedo and pulp samples were analyzed independently and are displayed in **a**) and **b**), respectively. Only the principal components PC1 and PC2 are shown. CHP: *C. maxima*, DIA: *C. medica*, EUR: *C. limon*, SCM: *C. reticulata*, SSO: *C. aurantium*, SWO: *C. sinensis*, WLM: *C. deliciosa*

RNA-Seq analysis yielded an average of 24 million reads per sample, 90% of which mapped to the 25,434 annotated transcripts from the clementine reference genome. A principal component analysis was performed based on transformed read counts (see methods), from pulp and flavedo samples. As expected, the three replicates from each sample clustered together in all cases, supporting the reproducibility of the results (Fig. S1). Pure species were the most different samples in both tissues, as indicated by their dispersion in the PCA plot (Fig. 2). In contrast, admixed samples, and most notably sweet and sour oranges, were scattered in between the ancestral pure species.

Hierarchical clustering of pulp samples grouped together lemon and citron, apart from the other species. A second cluster only included pummelo, and the third one grouped the two mandarins (pure and admixed), and the two oranges (sour and sweet). Similar results were obtained for flavedo samples (Fig. S1).

### Differentially expressed genes during fruit ripening

In order to find differentially expressed genes among samples, all versus all pairwise comparisons were performed, as well as between groups of samples based on their phenotype. The last ones yielded the most significant and informative results, so we focused our study on them. For example, the comparison of pulp from citron and lemon, highly acidic and with low sugar content, against the remaining species, retrieved 6681 DEGs, while 1329 DEGs were found when compared flavedo from red and yellow fruits (at least one two-fold expression change, s-value < 0.01). Roughly 20% of the DEGs corresponded to uncharacterized loci and genes of unknown function, matching the uncharacterized proportion of the total genic space of *Citrus clementina*. Four differentially expressed genes and the housekeeping gene *CitUBC1* were selected to perform RT-qPCR confirmation of the differential expression analysis results. Overall, the log fold changes obtained from the RNA-seq data matched those calculated via the ΔΔCt method (Table S1), with a Pearson correlation coefficient between RNA-seq fold changes and ΔΔCt of 0.91 and 0.89 in pulp and flavedo samples, respectively.

**Fig. 3:**
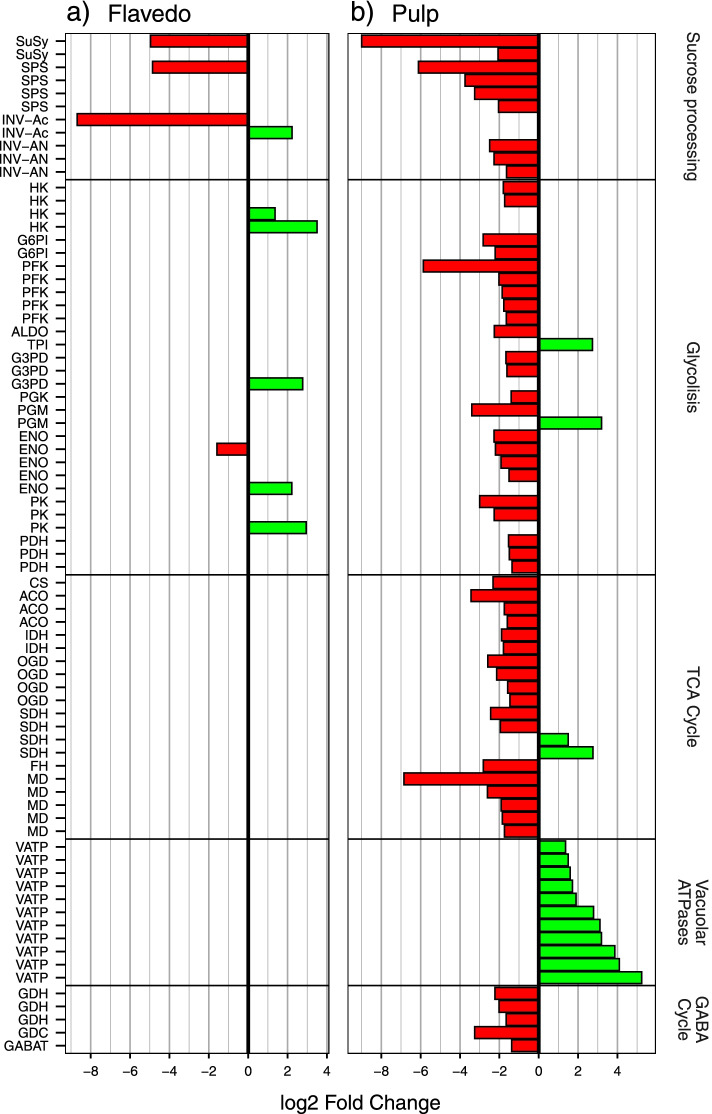
Differentially expressed genes involved in sugar metabolism. DEGs found in flavedo (**a**) and pulp (**b**) are shown independently. Each bar represents the expression log2 fold change comparing lemon and citron against the remaining samples (only genes with a log2 fold change > 1, s-value < 0.01, are shown). *SuSy: sucrose synthase, SPS: sucrose-phosphate synthase, INV − Ac: acid invertase, INV − AN: alkaline/neutral invertase, HK: hexokinase, G6PI: glucose-6-phosphate isomerase, PFK: 6-phosphofructokinase, ALDO: fructose-bisphosphate aldolase, TPI: triosephosphate isomerase, G3PD: glyceraldehyde-3-phosphate dehydrogenase, PGK: phosphoglycerate kinase, PGM: phosphoglycerate mutase, ENO: enolase, PK: pyruvate kinase, PDH: pyruvate dehydrogenase, CS: citrate synthase, ACO: aconitate hydratase, IDH: isocitrate dehydrogenase, OGD: 2-oxoglutarate dehydrogenase, SDH: succinate dehydrogenase, FH: fumarate hydratase, MD: malate dehydrogenase, VATP: V-type proton ATPase subunit, GDH: glutamate dehydrogenase, GDC: glutamate decarboxylase, GABAT: γ-aminobutyrate transaminase*

**Fig. 4:**
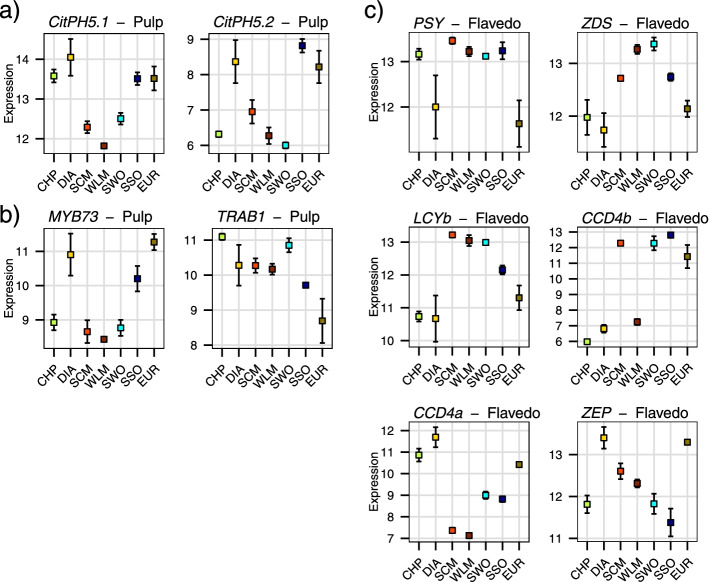
Expression levels of selected genes in each species. The selected genes are involved in **a**) pulp acidity, **b**) sugar accumulation or **c**) carotenoid accumulation. Dot colors represent different samples as in Fig. 2. CHP: *C. maxima*, DIA: *C. medica*, EUR: *C. limon*, SCM: *C. reticulata*, SSO: *C. aurantium*, SWO: *C. sinensis*, WLM: *C. deliciosa*

Differentially expressed genes associated with relevant metabolic pathways involved in fruit ripening were selected for further analyses: glycolysis / gluconeogenesis, tricarboxylic acid (TCA) cycle, vacuolar proton intake and secondary metabolism including carotenoid and flavonoid biosynthesis: a brief list of DEGs possibly involved in Citrus ripening were reported in Table S2. As citrus fruit taste mostly depends on citrate and sugar accumulation and the vacuolar pH of the pulp, we found interesting to compare the acid low-sugar fruits lemon and citron versus the remaining species (Fig. 1), and found that many genes involved in sugar metabolism and the TCA cycle consistently showed differential expression patterns. This way, most of the genes involved in these processes displayed lower expression levels in lemon and citron when compared to the remaining samples, including those genes involved in the TCA and the γ-aminobutyric acid (GABA) cycles, while most subunits of the vacuolar ATPase were overexpressed in these 2 species (Fig. 3). Besides these pathways, some specific genes previously related to sugar and citrate accumulation were analyzed. For example, two isoforms of *CitPH5*, a P-type ATPase involved in vacuolar acidification [22], were more expressed in the most acidic species, citron, lemon and sour orange, although similar expression levels were observed in pummelo in one of these genes (Fig. 4a). The expression of specific transcription factors regulating sugar and acid accumulation in other fruits [49–51] was also analyzed, and their levels in the different pulp correlated with sugar accumulation and titratable acidity (Fig. 4b). Functional annotation and enrichment analysis of the DEGs found in citron and lemon pulp samples revealed a significant enrichment in terms related with organic acid metabolism and ion transport via ATP hydrolysis (Table S3). We could not find these patterns in the flavedo samples, where the number of DEGs was considerably lower when compared to the pulp ones.

A two-way comparison of red (sweet and sour orange, pure and admixed mandarin) against yellow (lemon, citron and pummelo) colored varieties revealed only a few differentially expressed genes (Fig. S2). Indeed, comparisons between sample pairs yielded several differentially expressed genes of the carotenoid biosynthetic pathway, albeit not consistent across all the samples with the same fruit color (Fig. 4c). One of these genes encodes a *phytoene synthase* (*PSY*, LOC18039146), one of the first enzymes in the carotenogenic pathway, which was significantly downregulated in citron and lemon. The citrus ζ*-carotene desaturase* gene (*ZDS*, LOC112098231) was more expressed in red fruits than in yellow ones, although not all comparisons showed significant differences. Another gene coding for a key enzyme involved in the synthesis of *β*-*β-*carotenes, a *β-lycopene cyclase* (*LCYb2*, LOC18034834), was consistently overexpressed in all the red fruits compared to the yellow ones. Several genes coding for carotenoid cleavage dioxygenases (*CCDs*), involved in carotenoid accumulation and color setting, were also differentially expressed. One of them, *CCD4a* (LOC18043465), displayed lower expression levels in mandarins and oranges, while *CCD4b* (LOC18043103) was more expressed in sweet and sour oranges, lemon and pure mandarin. The *zeaxanthin epoxidas*e gene (*ZEP* LOC18036737), involved in carotenoid degradation, was overexpressed in lemon and citron. It should be noted that these comparisons were performed on flavedo samples since color break takes place earlier in the pulp, although pulp samples presented similar expression patterns for most of these genes.

**Fig. 5:**
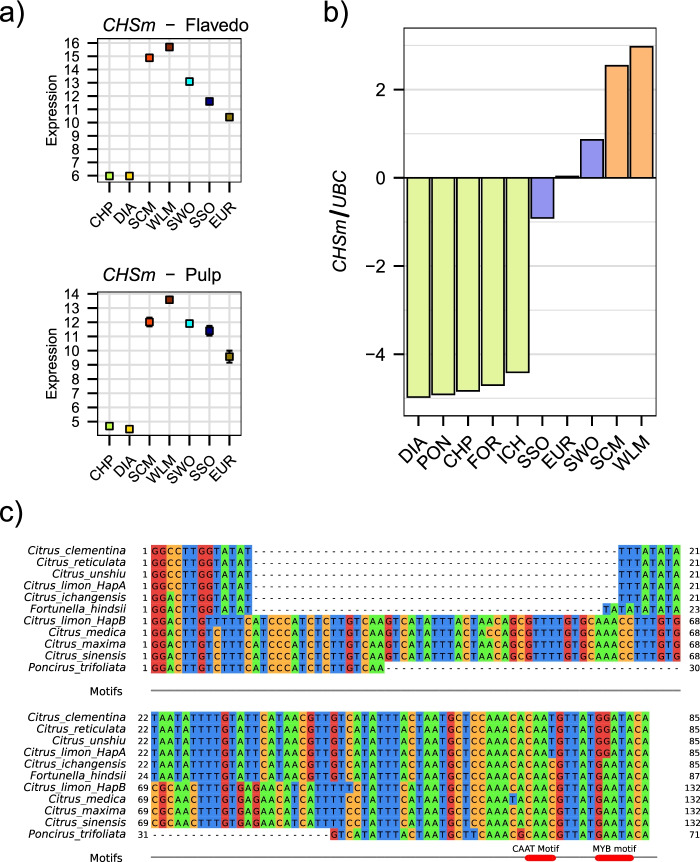
*CHSm* expression and promoter. **a** Expression levels of CHSm in pulp and flavedo deduced from RNA-seq data. **b** Expression levels of CHSm in flavedo calculated from RT-qPCR in the samples used for RNA-seq and other *Citrus* species. The color code represents the number of mandarin alleles of *CHSm* according to the admixture patterns published by Wu et al. (2018): green for zero mandarin alleles, blue for one allele and orange for two alleles. **c** Multiple sequence alignment of a fragment of the promoter region of CHSm. The CAAT and MYB motifs, and the region discriminating the structural alleles are highlighted. CHP: *C. maxima*, DIA: *C. medica*, EUR: *C. limon*, SCM: *C. reticulata*, SSO: *C. aurantium*, SWO: *C. sinensis*, WLM: *C. deliciosa*

Finally, the flavonoid biosynthetic pathway was studied in order to address the huge variability of flavonoid derivatives found in *Citrus* flavedo [19], that corresponds with the high number of genes involved in the production and regulation of these secondary metabolites [46]. Indeed, flavonoid diversity correlates with the extreme diversity of expression patterns observed in a large number of genes involved in flavonoid modifications (Fig. S3), especially the flavonoid and phenylpropanoid O-methyltransferases (FOMTs) families. Many of these genes had no expression in at least one species, while showing considerably high expression levels in others (Fig. S3). The most noticeable differential expression was displayed by a *chalcone synthase* gene *CHS* that was exclusively expressed at high levels in mandarin and their admixed species, lemon, sweet orange, sour orange and commercial mandarin (Fig. 5a). We will refer to this locus as *CHSm* due to their mandarin-linked expression, which was confirmed in both pulp and flavedo samples.

### Segmental ancestry patterns and differential gene expression

To assess the effects of the different introgressions in the global gene expression and domestication, the number of DEGs between the two palatable admixtures (sweet orange and commercial mandarin), and the pure species that originated them (pummelo and the pure mandarin) were analyzed. Using the admixed regions provided by Wu et al. [1], each gene was assigned to a specific admixture pattern based on their chromosome location, and the distribution of DEGs across the genome was studied.

**Fig. 6:**
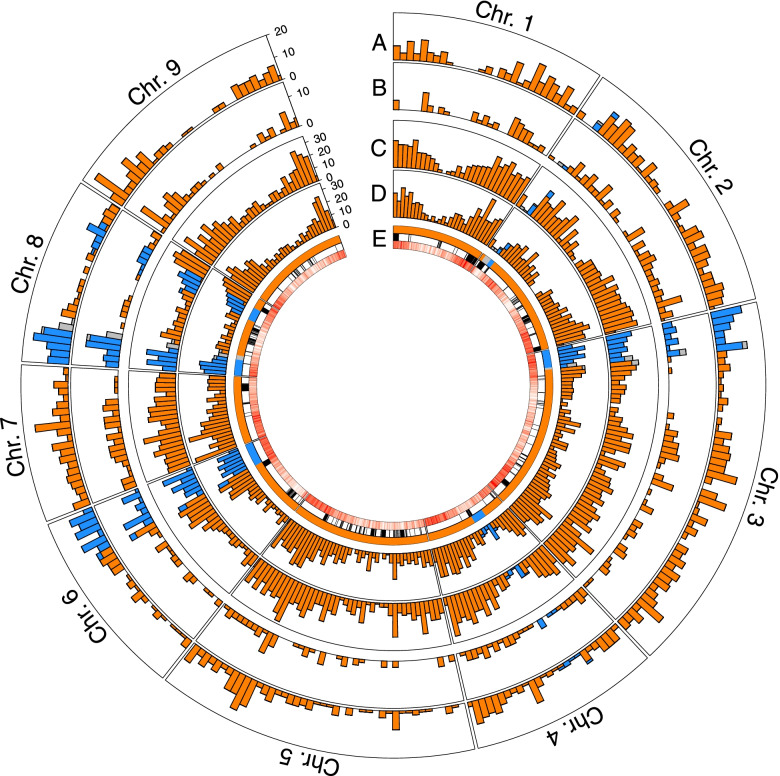
Distribution of DEGs between commercial mandarin and its progenitor species. The number of DEGs along the genome is represented in four different comparisons. Bar height represents the number of DEGs in a given windows, with color representing the admixture pattern of these genes (blue: admixed, orange: non-admixed mandarin). Bars with two colors represent genomic windows spanning two different admixed regions and include genes with both ancestries. **A**: Flavedo of wild and domesticated mandarin. **B**: Pulp of wild and domesticated mandarin. **C**: Flavedo of pummelo and domesticated mandarin. **D**: Pulp of pummelo and domesticated mandarin. **E**: admixture patterns of the commercial mandarin genome and distribution of runs of homozygosity (in black) and genic abundance (in red)

The number of genes differentially expressed between wild and domesticated mandarin was considerably higher on the pummelo/mandarin admixed regions of the genome than in the rest of it. This was especially evident at the end of chromosome 6 and the beginning of chromosomes 3 and 8 (Fig. 6). Among these DEGs, some could be directly related to ripening, such as the *malate dehydrogenase MDH* (LOC18034657), which was significantly more expressed in wild mandarin pulps, a flavanone rhamnosyltransferase (LOC18035124) highly expressed in pummelo and commercial mandarin, or the already mentioned *CCD4b*. Several transcription factors were also expressed at different levels between the two mandarins: 5 genes in the case of pulp samples, and 7 in the flavedo ones. In pulp, three out of the five differentially expressed transcription factors were related to vernalization, based on their similarity to the *vernalization gene VRN1* (LOC18035586, LOC18034082 and LOC18034239); two of them were also differentially expressed in the flavedo. In contrast, in the pummelo - commercial mandarin comparison, more DEGs were found along the whole genomic space, but the pummelo/mandarin admixed regions were not particularly enriched on these.

In sweet orange, the number of DEGs in the admixed regions was not remarkably different when comparing this cultivar with its ancestors, but this contrasts with the observation made in non-admixed regions of the genome. Specifically, the main pummelo/pummelo region of the sweet orange genome, located at the end of the chromosome 2, was amongst the regions with a higher amount of DEGs against mandarin (Fig. S4), while it was not particularly enriched when compared with pummelo. Conversely, the mandarin/mandarin regions of the sweet orange genome, and especially those at the end of the chromosomes 3 and 9, displayed a large amount of DEGs against pummelo, while showing considerably less when compared with mandarin (Fig. S4).

The number of DEGs was also studied across the existing runs of homozygosity on the commercial mandarin and sweet orange genomes. The runs of homozygosity, here defined as genomic windows with a heterozygosity below 0.1%, were scarce in sweet orange, with only 1% of its genome being composed by runs of homozygosity. In the case of the commercial mandarin, this percentage ascended to 11.4%. In none of the two species DEGs were particularly enriched in the runs of homozygosity.

### Allele-specific expression in *Citrus* ripening genes

In order to find allele-specific expression in the admixed genomic regions, the level of expression of each allele was assessed independently. Allele expression imbalance was calculated based on species-specific markers, diagnostic SNPs that allow phasing along the genome. This strategy can properly phase the two alleles in admixed species, but only in the genomic regions displaying the 2 ancestral haplotypes. As the extent of admixed regions differs greatly between species (from a mere 10% of the genome in commercial mandarin to the whole genome in lemon and sour orange), the total number of diagnostic SNPs was not directly comparable. However, the number of diagnostic SNPs per kb laid between 6 and 10 SNPs in the admixed region of every species (Table 1).

**Table 1:** Number of diagnostic SNPs and admixture proportions in four commercial *Citrus* cultivars

Sample	Diagnostic SNPs	Admixture proportion	Diagnostic SNPs per admixed kb
Sweet Orange	1,339,133	82.4%	5.62
Sour Orange	1,765,492	99.6%	6.13
Willowleaf Mandarin	194,888	9.3%	7.26
Eureka Lemon	2,171,266	98.1%	7.66

The phasing information was used to identify allele specific reads, so it was possible to quantify expression at the allele level, and therefore, to detect genes displaying allele-specific expression (ASE). In commercial mandarin, for instance, 380 genes displayed ASE in any of the two tissues: 99 were specific from flavedo, 160 from pulp, and 121 were common to both tissues. In lemon these numbers were considerably higher, with 1324 genes displaying ASE only in flavedo, 2021 only in pulp and 937 showing this behavior in both tissues. Despite the differences in the raw number of ASE genes, when considering the admixture proportions of each species, the number of genes displaying ASE was comparable with the exception of lemon, which displays a considerably higher number of ASE genes. For example, the number of ASE genes in the citron/mandarin admixed regions of lemon, which represent around 50% of the genomic space, was as high as that found in the pummelo/mandarin regions in the hybrid sour orange (Fig. S5). This was especially pronounced in pulp samples, that displayed more ASE genes than the flavedo ones in all the analyzed species. In the lemon pulp about 12% of the total genic content (2958 genes) displayed ASE, while it was around 8% in the case of sour orange (2232 genes) and the other two admixtures, where similar proportions were found.

Several genes showing allele-specific differential expression have relevant roles in the ripening process and might have been selected during the domestication process. For instance, the citron allele of one *hexokinase* gene (*HK*, LOC18035909) was preferentially expressed in lemon pulp, while in sweet orange the pummelo allele was the one overexpressed. The pummelo allele of a GABA transporter (LOC18035651), which might play a role in the GABA shunt, was preferentially expressed in commercial mandarin and sweet orange, while both the pummelo and mandarin alleles have similar expression levels in sour orange. Other examples include the abovementioned *phytoene synthase PSY* gene, for which the mandarin allele was preferentially expressed in sweet orange and commercial mandarin, and the *carotenoid cleavage dioxygenase 4 CCD4a*: in sweet and sour orange flavedo the pummelo allele was almost exclusively expressed, while in lemon the citron and pummelo alleles were expressed at comparable levels. Another relevant example is that of a *chalcone synthase CHSm*, in which only the mandarin allele was expressed in lemon, sweet orange and sour orange (Table S4), that was studied in detail.

### Promoter structure of the chalcone synthase *CHSm*

As described above, in the pure species, *CHSm* was only expressed in mandarin. In the admixed commercial varieties, only the mandarin allele was expressed, and the pummelo and citron alleles were hardly expressed. To study putative causes of this reduced expression in citron and pummelo, the promoter region of *CHSm* was studied. A 500 bp sequence upstream of the *CHSm* locus was retrieved from the *C. clementina* reference genome, and orthologs in other available citrus genome sequences, including two different lemon haplotypes recently published [2, 4, 15, 16, 52–55], see Methods. By aligning the 11 sequences, we found a number of differential SNPs that allow the discrimination of the species-specific alleles; a region of almost 100 bp with different sequences between pure species was also found (Fig. 5c, complete alignment in Fig. S6), with three different structural alleles. A motif search using the NewPLACE database revealed the existence of some sets of motifs that were uniquely found in the mandarin allele and shared by the mandarin admixtures but are absent in the other species (Table S5), most of which arise due to specific SNPs (Fig. 5c). The nucleotide sequence was confirmed in citron, pummelo and mandarin via Sanger sequencing (see Table S6 for primer details). The expression of *CHSm* in different Citrus species was verified via RT-qPCR, revealing that its high expression was exclusive to mandarins, while it was intermediate in the admixtures harboring a single mandarin allele and very low in the rest of pure species (Fig. 5b).

## Discussion

### Admixture patterns and their role in shaping gene expression

Recent genomic studies of commercial citrus species have shown extensive introgressions in their genomes from three pure species: citrons, pummelos and mandarins. The principal component analysis (PCA) based on genomic sequence variability depicted the three pure species in the vertices of a triangle, with the admixtures scattered somewhere between them [1]. A phenotype-based PCA study, using 146 citrus botanical traits produced similar results [56]. In our study, the PCA based on gene expression also generated a highly similar distribution (Fig. 2). It is worth noting that completely different types of the data gave rise to the same distribution, linking the genomic, transcriptomic and phenotypical levels. This observation suggests that indeed admixture patterns might have a relevant role in regulating gene expression that would result in the wide phenotypic variability found in citrus fruits.

It has been reported, that in plant interspecific hybrids, gene expression can reach extreme values when compared to both parentals, in a process called transgressive gene expression [57]. This phenomenon is explained by inter-loci epistatic relations and complementarity between loci, among others [58], and can sometimes result in improved phenotypes compared to the parental species [59]. However, our work shows that, in *Citrus*, admixed transcriptomes display a global behavior intermediate between their ancestor pure species, albeit transgressive expression can be found in specific genes or traits.

In hybrids of other plant crops, ASE has been described in specific genes [60, 61], possibly increasing the fitness of the hybrid species by providing higher genomic plasticity [62]. Within this frame, we studied the abundance of genes displaying ASE in the four admixed cultivars. Allele-specific expression was more prevalent in all the pulp samples when compared to the flavedo ones (Fig. S5), and slightly more common in lemon, with about 12% of the total gene number displaying allele-specific expression. This value is comparable with the results found in hybrid rice, in which nearly 6% of the genic space displays some sort of ASE [60]. However, other crops display higher proportions of ASE genes, such as tomato [63] or maize [64], reaching 20 and 50% of the total number of genes. Overall, ASE affects a low percentage of the genic space in *Citrus*, while quantitative changes in total expression affect a larger proportion of genes, suggesting that they might better explain phenotypic differences between cultivars.

To this end, we studied the distribution of differentially expressed genes along the genomes of the two palatable admixtures included in this study: the commercial mandarin and the sweet orange. DEGs between the commercial and the pure mandarin are considerably more concentrated in the admixed regions of the commercial mandarin genome, while the runs of homozygosity of the genome do not show any significant increase (Fig. 6). Interestingly, the start of chromosome 8 showed one of the highest accumulations of DEGs in both the pulp and the flavedo samples. A previous study proposed this region as a major domestication target for mandarins, based on the high prevalence of this introgression in commercial mandarins, as well as its significant association with fruit quality traits found in a genome wide association study [1]. Although the authors suggested an *isocitrate dehydrogenase* gene located on that region as a putative determinant of fruit acidity, we could not find differential expression of this gene between any two species. However, a *malate dehydrogenase* gene overexpressed in wild mandarin was identified in this region in our work. *MDH* has been previously associated with *Citrus* pulp acidity [65] and might have been a target of the domestication process. The start of chromosome 8 has been also associated with fruit weight [66], and a correlation between fruit size and the amount of pummelo introgression has been suggested [1]. The admixed region located near the end of chromosome 8 also displayed a flavanone rhamnosyltransferase which is differentially expressed in the two mandarins; this enzyme is involved in the bitterness of some citrus cultivars [67, 68] and therefore represent a putative domestication target. The beginning of chromosome 3, which is significantly associated with fruit firmness and easy peeling [66], also displayed a high amount of DEGs between wild and commercial mandarins, especially in flavedo.

Additional evidence supports the importance of these admixed regions of the genome in the process of *Citrus* domestication. For instance, three different loci coding for the vernalization transcription factor *VRN1* were differentially expressed between both mandarins. Very recently, *VRN1* expression has been related to the regulation of fruit ripening in *Pyris* [69], as it indirectly promotes the expression of many genes related with this process, having a pleiotropic effect. *VRN1* analogs have been also found differentially expressed in other fruit crops [70, 71], which again suggests a role of these transcription factors in orchestrating fruit ripening. In *Citrus*, domestication has targeted major transcription factor families [72], and furthermore, *VRN1* genes are located on the abovementioned admixed region of the beginning of chromosome 8. This could explain why this admixed region is conserved among most if not all commercial mandarins, while also being extensively linked with domestication [1, 54, 66]. Other relevant genes found in this admixed region also include a GABA transporter *GAT1* (LOC18035651), which is involved in the GABA intake from the apoplast [73] and could be related to citrate accumulation in citrus fruits [27]. Hence, the preferential expression of the pummelo *GAT1* allele in commercial mandarin pulp might be linked with the domestication process.

Comparison of cultivated vs. wild mandarins showed that the differentially expressed genes were more frequently located on the small fraction of P/M admixed regions of the genome. On the other hand, in sweet orange, that has a mostly admixed genome, the major differences at the expression level were found in the scarce non-admixed regions. This might suggest that the specific admixture pattern of each commercial citrus cultivar has a global effect at the transcriptomic level, which appears to be related to the results obtained in the principal component analysis (Fig. 2). Indeed, interspecific hybridizations represent a major force in the domestication of many tree species such as olives, apple or grapevine [74–76], as well as in some annual crops such as tomato or maize [8, 77]. Given the ease of vegetative propagation of *Citrus* and the apomictic nature of many commercial cultivars, the domestication process of these species might have started with the initial generation of specific admixture patterns by hybridization between a reduced number of individuals, thus generating a considerable degree of inbreeding [1]. From there, cultivars of interest were selected, maintaining specific segmental ancestry patterns that provided desirable traits [1, 72], and further producing new cultivars via selection of somatic mutants.

### Determinants on sugar and acidic contents among species

Gene expression analysis of acid fruits showed a large number of DEGs involved in carbohydrate metabolism in citron and lemon pulp (Fig. 3). Some of these genes coded for enzymes involved in hexose mobilization, like sucrose synthases (*SuSy*), sucrose phosphate synthases (*SPS*), sucrose phosphatases (*SPP*) and invertases (*INV-Ac* and *INV-AN).* Most of the sugars found in citrus fruits are actually synthetized elsewhere, and then translocated into them [78], and sugar sink strength has been linked to the hydrolysis of sucrose by several enzymes including *SuSy* [78, 79]. In other fruits, such as pear or apple, sugar accumulation has been also linked with hexokinase activity [80, 81]. In our study, sugar content remained invariably low across the whole ripening process in lemon and citron samples, which correlates with the reduced expression of the *SuSy* and *hexokinase* genes. Moreover, the latter also displayed ASE in lemon pulp, where the citron allele is preferentially expressed.

Interestingly, the expression of some specific transcription factors correlated with the accumulation of sugar in the pulp from some samples. For example, the transcription factor *TRAB1* (LOC18033660) had the lowest expression level in lemon pulp (Fig. 4b). *TRAB1* is a homolog of the *Malus domestica* gene *MdAREB2*, whose expression is related to an increased sucrose uptake, that causes a larger accumulation of soluble sugars, which in turn alters the fruit sink strength [50]. The reduced *TRAB1* expression in lemon fruits could partially explain the lower sugar accumulation, although it cannot explain the differences in citron, which might depend upon other mechanisms.

Another remarkable observation is the consistently lower expression in lemon and citron of several genes involved in carbohydrates and organic acids metabolism, while the expression of genes coding for several V-ATPase subunits was significantly increased (Fig. 3). Moreover, many differentially expressed genes in citron and lemon pulp are involved in organic acids metabolism and ATP-dependent molecular transport of several molecules (Table S3). Recently, the P-type ATPase CitPH5, which is located on the tonoplast membrane, was postulated as a relevant factor in determining the vacuolar proton gradient [22], while other studies report that V-ATPases can fulfill the same role, complementing each other [82, 83]. We found that one of the isoforms of *CitPH5* was indeed overexpressed in the three most acidic accessions, citron, lemon and sour orange (Fig. 4a), in agreement with previous results [22]; however, another *CitPH5* isoform was also highly expressed in pummelo, whose acidity is very low. The consistent overexpression of V-ATPases in citron and lemon pulp, coupled with the higher *CitPH5* P-ATPase activity, suggest that the two mechanisms might be working in these species, maintaining a high acidity throughout the whole ripening process (Fig. 1).

The study in detail of transcription factors associated with the regulation of citric acid content showed a MYB transcription factor, *CrMYB73*, that was overexpressed in citron, lemon and, to a lesser extent, in sour orange (Fig. 4b). *CrMYB73* is highly similar to the *Petunia hybrida PhPH4* gene, and is involved in the accumulation of citric acid in mandarin pulp over time [84]. Furthermore, it has been shown that the expression of *CrMYB73* is knocked out in some acidless sweet orange mutants [22]. Hence, our results suggest a relevant role of *CrMYB73* in the accumulation of acid in citron, lemon and sour orange.

Despite the evidence of some allelic imbalance affecting rate-limiting glycolytic enzymes in lemon pulp, our work indicate that the differential expression of genes involved in many steps of sugar metabolism might be the main cause of the differences in acid contents in fruit. We present evidence supporting that *CitPH5* is relevant for citrus acidity, since it is certainly overexpressed in the three most acidic samples: citron, lemon and sour orange (Fig. 4a). However, our data also suggest the possibility of an additional mechanism besides *CitPH5*, that would involve the accumulation of citrate in the pulp of citron and lemon by an increased V-ATPase activity (Fig. 3). This is likely the result of the complex interplay among the transcription factors analyzed above, and very possibly many others not studied in this work. The overall reduction in sugar accumulation in these fruits would also contribute to increase their sourness.

### Citrus pigmentation from a genus-wide perspective

We observed several changes in the expression of multiple key genes involved in carotenoid biosynthesis (Fig. S2), suggesting that citrus color might not depend on a single master gene, but instead on the additive effects of several genes (Fig. 4c). For example, a *PSY* gene, whose expression in some citrus has been shown to correlate with total carotenoid content [85, 86], was downregulated in citron and lemon fruits. The total carotenoid content of these fruits has been thoroughly characterized in previous studies [87, 88] showing that the carotenoid content of yellow fruits such as lemons, citrons and pummelos are lower than those of red-peeled varieties such as mandarins or oranges. We did not find the reduction of *PSY* expression in pummelo, which also produces yellow fruits, suggesting that a different mechanism might be involved in the low carotenoid content described in this species.

Another differentially expressed gene coding for a ζ*-carotene desaturase* was overexpressed in the flavedo of sweet orange and the commercial mandarin, both producing red fruits. *ZDS* is essential for the red coloration of tomatoes, as it is required for the production of lycopene and β-carotene derivatives [89]; *ZDS* has been also associated with carotenoid biosynthesis in carrot [90]. In *Citrus*, the sweet orange Pinalate mutant, that produces yellow fruits, was initially thought to be a *ZDS* defective mutant [91], linking *ZDS* activity with the red coloration of sweet oranges. Further studies revealed that the defective gene was a *ζ-carotene isomerase*, and not a desaturase [92]. The high expression levels of *ZDS* gene that we found in sweet oranges and commercial mandarin might suggest that *ZDS* might after all be involved in the red pigmentation of oranges and mandarins.

One of the main branching points in the carotenoid biosynthesis pathway is the lycopene cyclization, carried out by the *lycopene β-cyclase LCYb*, which funnels the carbon flux towards the *β*-*β-*carotene production [36]. Actually, *LCYb* gene expression has been shown to increase during color break of mandarin and orange fruits, suggesting its role in this process [87, 93]. In this work we show that the *LCYb2* gene was consistently overexpressed in the flavedo of all red fruits, when compared to the yellow ones from citron, pummelo and lemon, both in the pairwise and in the red against yellow fruits comparisons (Fig. S2). The role of *LCYb2* directing the carbon flux of the carotenoid pathway towards *β*-carotene and its derivatives has already been suggested in *Citrus* [36, 37] and other species such as carrot [94]. Overall, our results suggest that *LCYb2* activity might be involved with the fruit red coloration in different species from the genus *Citrus*, as its expression is consistently higher in red-colored peels.

The gene coding for a zeaxanthin epoxidase also presented differential expression patterns, being overexpressed in citron and lemon when compared with the remaining species, although only in pummelo, sweet orange and sour orange were statistically significant. In Arabidopsis, *ZEP* defective mutants accumulate *β*-carotene, *β*-cryptoxanthin and zeaxanthin due to a metabolic blockage in carotenoid degradation [95]; a similar observation was made in potato, where reduced *ZEP* expression resulted into the accumulation of zeaxanthin [96]. In maize, specific *ZEP* alleles have been identified as reliable predictors of total carotenoid content, highlighting their crucial role in this process [97]. According to our results, the increased expression of the *ZEP* gene we found in citron and lemon could be related with the lower carotenoid accumulation previously reported in the flavedo of these species [35].

We also found significant alterations in the expression of genes coding for carotenoid cleavage dioxygenases, including *CCD4b*, that has been postulated as the major enzyme involved in the production of the predominant red carotenoids in mandarins and oranges by cleaving *β*-carotene, *β*-cryptoxanthin and zeaxanthin into C30-apocarotenoids [98, 99]. Among our samples, *CCD4b* was significantly overexpressed in some but not all the red-colored flavedo samples. Commercial mandarin presented a low *CCD4b* expression, with levels comparable to those of the yellow citron and pummelo, while its expression in the lemon flavedo was similar to that of red fruits. *CCD4a*, a paralog of *CCD4b* [99], was differentially expressed among red and yellow species: it was hardly expressed in mandarins, displayed an intermediate expression in oranges and was highly expressed in the yellow fruits from pummelo, citron and lemon. Moreover, the ASE analysis performed in the present work showed that, in the flavedo of the admixed cultivars sweet and sour orange, the pummelo allele was expressed significantly higher than the mandarin one (over 90% of the expression came from the pummelo allele in both cases), while in lemon both citron and pummelo alleles were expressed at comparable levels. This may explain the intermediate expression level in sweet and sour orange, which might be produced by the combination of a highly expressed pummelo allele and an almost non-expressed mandarin allele. *CCD4a* has been considerably less studied in *Citrus* since its expression in mandarin and orange peel is low [98], but recent studies have reported that it is actually expressed in the flavedo of yellow fruits [100]. *CCD4a* is involved in the degradation of colored carotenoids in *Chrysanthemum* and *Petunia* petals [101–103], where an impairment in its expression results in an accumulation of carotenoids in the flower petals. Considering previously reported quantifications of the carotenoid content in peel from different *Citrus* species [87, 88], the postulated role of *CCD4a* in other species [101–103] and our results regarding its expression pattern in different cultivars, we propose that the differences in the expression of *CCD4a*, which appears to be subject to ASE, might be related to the yellow pigmentation of citrus peel.

Our results would not support the existence of a master gene controlling carotenoid accumulation, but rather suggest that this trait would depend on the additive effects of several genes, with some playing a more determinant role in peel pigmentation. For instance, we found that some catabolic genes, such as *CCD4a* and *ZEP*, are more expressed in citron, lemon and pummelo, whose peel reach lower overall carotenoid concentrations [87, 88]. Therefore, the increased catabolic activity might be involved in the lower carotenoid content found in these fruits. We also observed an increased expression of the *LCYb2* gene in all the red-colored cultivars. Earlier studies have suggested that an increased *LCYb2* expression might funnel the carbon flux towards the *β*-*β-*carotenoid branch [36], ultimately increasing the final carotenoid content. Our results also reveal that some key genes found upstream of the carotenoid biosynthetic pathways, including *PSY* or *ZDS,* are differentially expressed among samples. Since other authors suggested the importance of substrate availability in determining the final carotenoid profile in *Citrus* [98, 104], we believe that the expression changes in *PSY* or *ZDS* reported in our work might be also affecting it. The idea that citrus peel pigmentation depends on many independent genes has been already postulated [87], and is vastly supported by the large number of somatic mutants that display an altered fruit color, mostly due to mutations affecting genes in the carotenoid biosynthetic pathway [92, 105–108].

### Stepwise evolution of flavonoid accumulation profiles in mandarins

Citrus flavonoids have been thoroughly studied by other authors, as citrus peels display an huge range of flavonoids and flavonoid derivatives [19]. The flavonoid profiles are characteristic of each *Citrus* species to the point that clustering based on such profiles, reproduce their phylogenetic relationships, with mandarins and oranges clustering closely and being further away from other species [109]. This result was confirmed in another work that showed that mandarins and oranges accumulate the largest amounts of flavonoids [110], and specially O-methylflavonoids [109]. One of the first steps in the flavonoids biosynthetic pathway is the synthesis of naringenin chalcone, which is carried out by a chalcone synthase *CHS* [111]. This is a rate-limiting enzyme that acts as a major regulatory step in flavonoid production, as has been described in several plants including *Citrus* [112, 113]. In our work we found a chalcone synthase gene *CHSm* which was solely expressed in pure mandarin and mandarin derived species (Fig. 5a). ASE analysis revealed that only the mandarin allele was expressed in sweet and sour orange, as 99% of the reads were from the mandarin haplotype, indicating that the pummelo allele was silenced. The case of lemon is more complex: the genomic coordinates of the *CHSm* locus were previously assigned to a citron/pummelo (C/P) admixed region, hence a mandarin allele should not be present, as the closest citron/mandarin (C/M) admixed region is located about 60 kb away from *CHSm* locus [1]. However, a manual analysis based on diagnostic SNPs in the *CHSm* locus revealed that only the mandarin allele was expressed in lemon for that locus, which was further confirmed in the genomic sequencing and agrees with the haplotypes reported in the assembled lemon genome [4]. The assignment of this region as a citron/pummelo admixed region might be erroneous, possibly due to the proximity of a true citron/mandarin region, especially considering that the methodology used to determine the segmental ancestry in *Citrus* is more error-prone near admixed region boundaries, where the local ancestry can be ambiguous [1].

The analysis of the promoter region of *CHSm* revealed the existence of species-specific alleles which could be linked with the differences at the expression level of this locus. Such differences might result from specific SNPs in the promoter region, and indeed the mandarins, which are the only species here studied that express *CHSm* at high levels, presented some exclusive regulatory target sites, remarkably a MYB binding site and a CAAT box separated by five base pairs. MYB transcription factors are essential regulators of *CHS* expression in other species within the genus *Malus* [114], and indeed a MYB binding site is required for the expression of a different *chalcone synthase* gene in *Citrus* [115]. In turn, CAAT boxes have been recurrently found in the promoter region of *CHS*, in some cases near a MYB binding site, in many different species, including tobacco [116] and eggplant [117], among others [118, 119]. Therefore, we hypothesize that the mandarin directed *CHSm* expression depends on specific point mutations on the promoter region of the gene, possibly including the existence of a MYB binding site and a CAAT box, albeit the different promoter structures that distinguish the mandarin, pummelo and ancestral structures may be also playing a role in this regulation.

As *CHS* is a rate-limiting enzyme in the flavonoid biosynthetic pathway, an increased expression of *CHSm* could lead to a greater flavonoid accumulation, as previously reported in *Citrus* [113]. Notably, the study of the expression of the genes involved in the flavonoid biosynthetic pathway revealed a significant variability in the expression of flavonoid-modifying enzymes (Fig. S3), and especially of flavonoid-O-methyltransferases. Methylated flavonoids, and particularly, polymethoxylated flavonoids, are a diverse family of compounds fulfilling multiple biological functions. Previous studies have assigned a broad substrate specificity to *Citrus* FOMTs [120, 121], whose expression is also extremely variable in different tissues and development stages, with up to 58 different genes with different expression patterns [122]. The recent expansion of FOMT gene family in *Citrus* compared to other plant lineages, much more prominent in the case of mandarins [46], might be related this expression variability. It is well known that gene family expansion paves the way for neofunctionalization by providing with extra copies of genes belonging to the family, therefore allowing for a better adaptation to new environments [123].

It is interesting to note that the FOMT gene family is specifically expanded in mandarins [46], which have gone through an intensive domestication process. The increased *CHSm* expression we report could in principle generate greater amounts of flavonoid precursors, which could be further modified by the expanded FOMT family to produce a broader range of compounds. Considering that the FOMT expansion is by far the largest in mandarins [72], it appears plausible that the mandarin *CHSm* allele activity, by generating a higher abundance of substrate, could be one of the factors leading to the expansion of the FOMT families, possibly during the mandarin early domestication or sometime in between the evolutionary and domestication processes.

## Conclusions

In this work we have performed a genus wide RNA-seq analysis in ripening fruits from wild and domesticated citrus species, in order to assess the impact on gene expression of the complex evolutionary and domestication histories of the commercial citrus varieties in gene expression during ripening. We report for the first time the effects of the segmental ancestry of specific citrus species in the expression patterns of the genes contained within, highlighting the importance of introgressions during the early domestication of the genus *Citrus*. The broad scope of our work provides an evolutionary viewpoint on *Citrus* ripening transcriptomics, a perspective of special interest in a genus where most commercial cultivars are the product of interspecific hybridizations.

## Methods

### Plant material

Plant material for the RNA-seq analysis was provided by the germplasm resources at the Instituto Valenciano de Investigaciones Agrarias (IVIA). Three pure species, Sun Chu Sha Kat mandarin (*Citrus reticulata*), Chandler pummelo (*Citrus maxima*) and Diamante citron (*Citrus medica*), and four admixtures, Seville sour orange (*Citrus aurantium*), Salustiana sweet orange (*Citrus sinensis*), Willowleaf mandarin (*Citrus deliciosa*) and Eureka lemon (*Citrus limon*) were analyzed.

Sun Chu Sha Kat is a type 1 mandarin that produces small acidic fruits with bright red peels. Chandler pummelo produces large round fruits with pale yellow peel, pink pulp and sweet taste. Diamante citron also produces large fruits, but with an ovate shape, and its pulp is yellow and much more acidic. These three cultivar names are abbreviated as SCM, CHP and DIA in the figures of this manuscript.

The Seville sour orange is a direct hybrid between a mandarin and a pummelo [2], displaying the two complete parental haplotypes. Sour orange fruits are extremely acidic, with bright red peel and medium size. Salustiana sweet orange produces medium-sized round fruits with low acidity and a red rind; it is a pummelo/mandarin admixture displaying M/M, P/M and P/P regions in its genome, corresponding to 14, 83 and 3% of the total genomic space respectively [2]. Willowleaf mandarin is a type 2 mandarin, which produces edible pale-red fruits of intermediate size. As a type 2 mandarin, it has pummelo introgressions, in this case accounting for roughly 9% of the genome, while the rest displays two mandarin haplotypes [2]. The Eureka lemon is a tri-specific hybrid in which 55% of its genome has citron/mandarin ancestry while the other 45% is citron/pummelo [1]. It produces medium-sized fruits with a yellow peel and extremely acidic pulp. These cultivars have been respectively abbreviated as SSO, SWO, WLM and EUR in the figures.

Fruit samples of Ichang papeda (*Citrus ichangensis*), the Nagami kumquat (*Fortunella margarita*) and *Poncirus trifoliata* were also collected from the IVIA germplasm bank. *Citrus ichangensis* produces small inedible fruits containing acrid oil droplets in their pulp. Kumquat fruits are very small, with a red rind and highly acidic. Finally, *Poncirus* produces medium yellow fruits, also inedible. The abbreviations used for these species are ICH, FOR and PON, respectively. Accession numbers of each cultivar and species used in this work are shown in Table S7.

### Phenotypical data collection

Fresh fruit samples were collected every three weeks, from mid-September to January. Peel color was measured on field using a hand colorimeter Konica Minolta CR400. For each sample analyzed, color was measured in four different fruits performing three technical replicates on each. Fruits were then collected and processed the same day.

Fruits were squeezed and the titratable acid content of the juice was measured by titration with 0.1 M sodium hydroxide and a phenolphthalein indicator. Juice total soluble sugar content was measured in Brix degrees using a table refractometer ATAGO PR-1. Brix and acidity were analyzed on the pooled juice performing three technical replicates.

### RNA extraction, library preparation and sequencing

For each species, three biological replicates were collected at different dates to match each species color break, in order to collect samples in a comparable physiological state. Flavedo and pulp tissues were manually separated from each fruit and treated independently. Tissues were grinded frozen and total RNA was extracted using the acid phenol extraction coupled with lithium chloride precipitation as described in Ecker 1987 [124]. RNA-seq library preparation and sequencing were carried out by Novogene Company. Briefly, RNA samples were enriched in mRNA using oligo (dT) beads and the mRNA was randomly fragmented. cDNAs were then synthesized from mRNA using random hexamers, followed by adapter ligation, size selection and PCR enrichment. Samples were sequenced in a NovaSeq 6000 platform, delivering 150 bp pair ended reads with an insert size of approximately 250 bp.

### RNA-seq read mapping and DEG analysis

Illumina reads were mapped against the *Citrus clementina* reference genome [2] using STAR 2.7.2 [125]. *C. clementina* genome annotation was downloaded from the NCBI and reads mapped to each genomic feature were counted using featureCounts 2.0 [126]. Read counts were normalized using a variance stabilizing transformation implemented in R [127]; these pseudocounts were used for the sample clustering for quality control and downstream analysis. Differential gene expression analyses were performed using the R package DESeq2 1.26 [128] following the author’s recommendations. Pulp and flavedo data were analyzed independently, performing pairwise comparisons among every species pair, as well as pairwise comparisons of grouped samples of citron and lemon against the rest and citron, lemon and pummelo against the rest. Differentially expressed genes (DEG, log2 fold change expression > 1, false sign or smaller rate < 0.01) were detected using the model implemented in apeglm [129]. Genes annotated into admixed regions [1] were used to assess the admixture effect in gene expression.

### KEGG enrichment analysis

A GO enrichment analysis was carried out for the comparison of citron and lemon pulp against the other analyzed samples. GO enrichment was performed using the R package clusterProfiler, KEGG data was accessed using AnnotationHub [130, 131]. To account for multiple hypothesis testing, *p*-values were corrected using the Benjamini-Hochberg method (FDR < 0.05).

### Confirmation of RNA-seq data by RT-qPCR

To validate the RNA-seq analyses and to assess the *CHSm* expression in other species, one-step RT-qPCR of a set of genes was carried out. Reverse transcription was performed by incubating the RNA samples with the reverse transcriptase MultiScribe (Invitrogen) at 48 °C, 30 min. RNAse activity was inhibited using RNAse Inhibitor (Applied Biosystems). Real-time qPCR was performed using the LightCycler FastStart DNA Master Plus SYBR Green I kit in a LightCycler 2.0 Instrument. Two technical replicates were performed for each reaction. Amplification specificity was verified by the presence of a single peak in the melting curve analysis. Oligonucleotides used for each reaction can be found in the Table S6.

Relative quantification of the gene expression was expressed as a log 2-fold change expression compared with a housekeeping gene, *CitUBC1* [132], using the ΔΔCt method.

### DNA extraction, sequencing and mapping

To find diagnostic SNPs and further validate the genomic structure of the studied genes, whole genome sequencing data was used. For the already published data, raw reads were retrieved from the Sequence Read Archive database (the SRA accession numbers are available in Table S7). The Diamante citron genome was sequenced in this work using Illumina whole genome sequencing. In short, high molecular weight DNA was extracted using an in-house protocol. Whole genome sequencing library preparation and sequencing were carried out by the Centro Nacional de Análisis Genómico (CNAG). Briefly, libraries were constructed using the Illumina TruSeq DNA Sample Prep protocol, selecting for an insert size of 500 bp. Paired-end sequencing was performed on a HiSeq 2000 instrument.

### Allele-specific expression

Allele-specific expression for each gene was studied with the following workflow: first, the two phases of every gene were established based on DNA sequencing using diagnostic heterozygous SNPs. Then, the mapped RNA-seq reads were scanned and those displaying different alleles of each diagnostic SNP were counted independently, which allowed the expression quantification at the allele level.

To achieve this, the Illumina genomic DNA reads (accession numbers available at Table S7) were mapped to the clementine reference genome using bwa mem [133] to generate one BAM file per sample, and SNPs were called in each sample using GATK 4.1.1 HaplotypeCaller in GVCF mode [134]. SNPs were hard-filtered following GATK best practices, and only SNPs showing a genotype quality (GQ) over 20 were selected.

Since pure species in *Citrus* show low heterozygosity, the two pseudophases could not be established based on phased SNPs. For admixed varieties, the admixture pattern of each genomic region was retrieved from previous works [1]. Then, for each gene within an admixed region, heterozygous SNPs were selected. Among them, diagnostic SNPs were defined as those sharing one allele with each pure species contributing to the admixture, being that species homozygous in that specific position. For example, an A/T position in sour orange would only be considered diagnostic if the two progenitor species, pummelo and wild mandarin, were A/A and T/T or vice-versa for that specific position. Diagnostic SNPs were used for allelic phasing as previously described [1, 2], generating one phase for each pure species analyzed. Finally, the number of RNA-seq reads mapped from each phase was counted using the samjdk utility of the jvarkit toolset [135].

### Detection of runs of homozygosity

The distribution of highly homozygous regions in the genome was studied to assess the prevalence on inbreeding in the palatable admixtures. To do so, all the heterozygous SNPs were retrieved from each genome, using the SNP set generated in the above section. The reference genome was split into non-overlapping 200 kb windows and those displaying a heterozygosity below 0.1% (less than 1 SNP per kb) were considered as runs of homozygosity.

### Analysis of the *chalcone synthase* promoter region

The upstream region of a *chalcone synthase* (*CHSm*, LOC18042808) was extracted from the *C. clementina* reference genome based on its genomic coordinates. The ortholog regions in the reference genomes of other *Citrus* species and related genera were obtained by similarity search using BLASTN 2.7.1 [136], comparing this sequence against publicly available assembled genomes of other *Citrus* and related species. The other genomes used correspond to citron (*C. medica*), pummelo (*C. maxima*), wild mandarin (*C. reticulata*), satsuma mandarin (*Citrus unshiu*), sweet orange (*C. sinensis*), two different lemon haplotypes (*C. limon*), Ichang papeda (*C. ichangensis*), the Hong Kong kumquat (*F. hindsii*) and, as an outgroup of the genus *Citrus*, *P. trifoliata* [2, 4, 15, 16, 52–55]. The ortholog sequence was verified in pure mandarin, citron and pummelo by conventional PCR using the forward and reverse primers described in Table S6. Each PCR product was sequenced using Sanger sequencing, and the sequences obtained were aligned using MAFFT [137]. The genomic structure of the region was also manually curated with the Integrative Genome Viewer (IGV) browser software [138] with the same DNA-based alignments used for allele phasing. After determining the sequence of the promoter region, the existence of species-specific promoter motifs was estimated using the online motif-detection tool NewPLACE [139].

## Supplementary Information

**Additional file 1: Figure S1.** Hierarchical clustering analysis. Hierarchical clustering of a) flavedo and b) pulp samples according to RNA-seq data. CHP: *C. maxima*, DIA: *C. medica*, EUR: *C. limon*, SCM: *C. reticulata*, SSO: *C. aurantium*, SWO: *C. sinensis*, WLM: *C. deliciosa*. **Figure S2.** Differentially expressed genes involved in carotenoid biosynthetic pathway. DEGs found in flavedo (a) and pulp (b) are shown independently. Each bar represents the expression log2 fold change comparing red samples (wild and domesticated mandarin, sweet and sour orange) against the yellow samples (lemon, citron and pummelo). Only genes with a log2 fold change > 1, s-value < 0.01, are shown. *ZDS: zeta-carotene desaturase, LUT5: beta-ring hydroxylase, LCYb: beta-lycopene cyclase, CHYB: carotenoid beta-ring hydroxylase, CCD4a: carotenoid cleavage dioxygenase 4a, AAH: abscisic acid hydroxylase*. **Figure S3.** Flavonoid-related gene expression across samples. Expression levels of genes involved in flavonoid modifications in flavedo tissues per sample and gene. Color intensity represent expression levels based on normalized read counts. Black rectangles mark differentially expressed genes between at least two samples; red rectangles denote flavonoid O-methyltransferases. CHP: *C. maxima*, DIA: *C. medica*, EUR: *C. limon*, SCM: *C. reticulata*, SSO: *C. aurantium*, SWO: *C. sinensis*, WLM: *C. deliciosa*. **Figure S4.** Distribution of DEGs between sweet orange and its progenitor species. The number of DEGs along the genome is represented in four different comparisons. Bar height represents the number of DEGs in a given windows, with color representing the admixture pattern of these genes (blue: admixed, orange: non-admixed mandarin, green: non-admixed pummelo). Bars with two colors represent genomic windows spanning two different admixed regions and include genes with both ancestries. A: Flavedo of sweet orange and wild mandarin. B: Pulp of sweet orange and wild mandarin. C: Flavedo of pummelo and sweet orange. D: Pulp of pummelo and sweet orange. E: admixture patterns of the sweet orange genome and distribution of runs of homozygosity (in black) and genic abundance (in red). **Figure S5.** Allele-specific expressed genes across tissues and cultivars. Total ASE genes preferentially expressing the citron, pummelo and mandarin in each sample, shown in green, orange and yellow, respectively. For each sample, regions with different ancestry were considered independently: M/P corresponds to mandarin/pummelo regions, C/P to citron/pummelo regions and C/M to citron/mandarin regions. The total number of ASE genes is shown on top of each bar. Flavedo (a) and pulp (b) samples are shown independently. CHP: *C. maxima*, DIA: *C. medica*, EUR: *C. limon*, SCM: *C. reticulata*, SSO: *C. aurantium*, SWO: *C. sinensis*, WLM: *C. deliciosa*. **Figure S6.** CHSm promoter sequence. Nucleotide sequence of 500 bp upstream from the gene start in 11 publicly available assembled genomes.

**Additional file 2: Table S1.** Results of the RNA-seq validation via RT-qPCR. **Table S2.** NCBI locus identifiers for the major genes discussed in this work. **Table S3.** GO Enrichment Analysis on the differentially expressed genes between citron and lemon and the remaining samples. **Table S4.** Allele-specific expression genes in metabolic pathways associated with ripening. **Table S5.** Regulatory motifs of the CHSm promoter. **Table S6.** Primers used for the validation of RNA-seq data and amplifying the CHSm promoter. **Table S7.** Sample information and origin.

## Data Availability

All data used and generated in this work is publicly available. RNA-seq raw reads were deposited under BioProject PRJNA785525, and the whole genome sequencing of *Citrus medica* was deposited under BioProject PRJNA784926. Prior resequencing data used in this work can be accessed under BioProjects PRJNA414519 and PRJEB5963. See Table S7 for more detailed information.
