# Supplementary material for: Comparative transcriptomics of wild and commercial Citrus during early ripening reveals how domestication shaped fruit gene expression
Source: BMC Plant Biol. 2022 Mar 17;22:123. doi: 10.1186/s12870-022-03509-9 (PMC8928680; doi:10.1186/s12870-022-03509-9)
Supplement: Supplementary file 1 — Additional file 1: Figure S1. Hierarchical clustering analysis. Hierarchical clustering of a) flavedo and b) pulp samples according to RNA-seq data. CHP: C. maxima, DIA: C. medica, EUR: C. limon, SCM: C. reticulata, SSO: C. aurantium, SWO: C. sinensis, WLM: C. deliciosa. Figure S2. Differentially expressed genes involved in carotenoid biosynthetic pathway. DEGs found in flavedo (a) and pulp (b) are shown independently. Each bar represents the expression log2 fold change comparing red samples (wild and domesticated mandarin, sweet and sour orange) against the yellow samples (lemon, citron and pummelo). Only genes with a log2 fold change > 1, s-value < 0.01, are shown. ZDS: zeta-carotene desaturase, LUT5: beta-ring hydroxylase, LCYb: beta-lycopene cyclase, CHYB: carotenoid beta-ring hydroxylase, CCD4a: carotenoid cleavage dioxygenase 4a, AAH: abscisic acid hydroxylase. Figure S3. Flavonoid-related gene expression across samples. Expression levels of genes involved in flavonoid modifications in flavedo tissues per sample and gene. Color intensity represent expression levels based on normalized read counts. Black rectangles mark differentially expressed genes between at least two samples; red rectangles denote flavonoid O-methyltransferases. CHP: C. maxima, DIA: C. medica, EUR: C. limon, SCM: C. reticulata, SSO: C. aurantium, SWO: C. sinensis, WLM: C. deliciosa. Figure S4. Distribution of DEGs between sweet orange and its progenitor species. The number of DEGs along the genome is represented in four different comparisons. Bar height represents the number of DEGs in a given windows, with color representing the admixture pattern of these genes (blue: admixed, orange: non-admixed mandarin, green: non-admixed pummelo). Bars with two colors represent genomic windows spanning two different admixed regions and include genes with both ancestries. A: Flavedo of sweet orange and wild mandarin. B: Pulp of sweet orange and wild mandarin. C: Flavedo of pummelo and sweet orange. D: P [file 12870_2022_3509_MOESM1_ESM.docx]

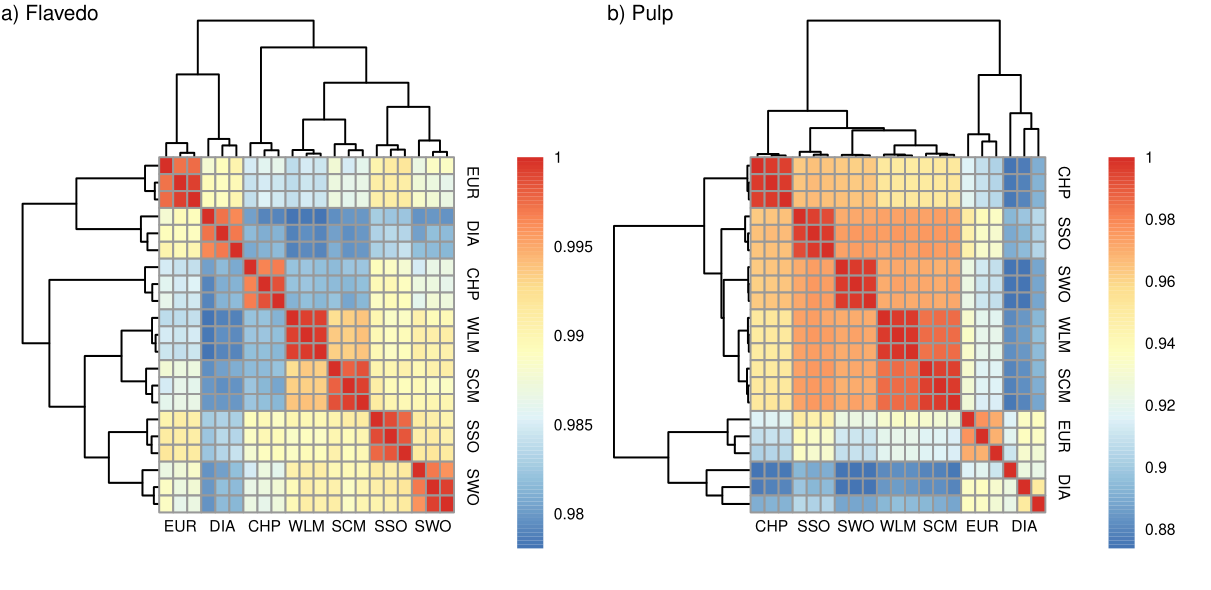
**Figure S1: Hierarchical clustering analysis.** Hierarchical clustering of a) flavedo and b) pulp samples according to RNA-seq data. CHP: *C. maxima*, DIA: *C. medica*, EUR: *C. limon*, SCM: *C. reticulata*, SSO: *C. aurantium*, SWO: *C. sinensis*, WLM: *C. deliciosa*.


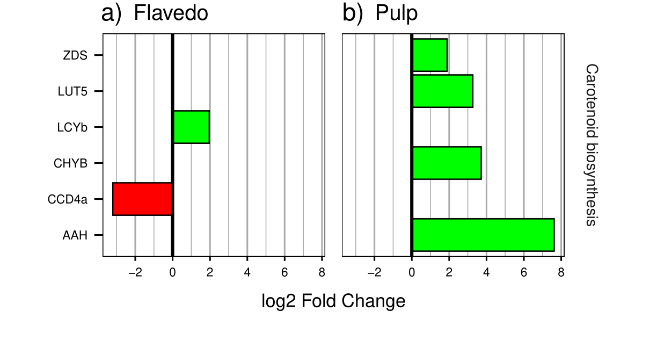


**Figure S2: Differentially expressed genes involved in carotenoid biosynthetic pathway.** DEGs found in flavedo (a) and pulp (b) are shown independently. Each bar represents the expression log2 fold change comparing red samples (wild and domesticated mandarin, sweet and sour orange) against the yellow samples (lemon, citron and pummelo). Only genes with a log2 fold change > 1, s-value < 0.01, are shown. *ZDS: zeta-carotene desaturase, LUT5: beta-ring hydroxylase, LCYb: beta-lycopene cyclase, CHYB: carotenoid beta-ring hydroxylase, CCD4a: carotenoid cleavage dioxygenase 4a, AAH: abscisic acid hydroxylase*.


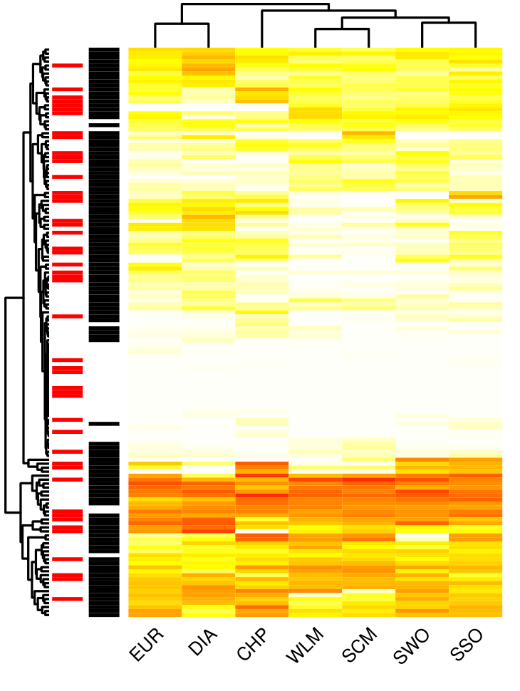


**Figure S3: Flavonoid-related gene expression across samples.** Expression levels of genes involved in flavonoid modifications in flavedo tissues per sample and gene. Color intensity represent expression levels based on normalized read counts. Black rectangles mark differentially expressed genes between at least two samples; red rectangles denote flavonoid O-methyltransferases. CHP: *C. maxima*, DIA: *C. medica*, EUR: *C. limon*, SCM: *C. reticulata*, SSO: *C. aurantium*, SWO: *C. sinensis*, WLM: *C. deliciosa*.


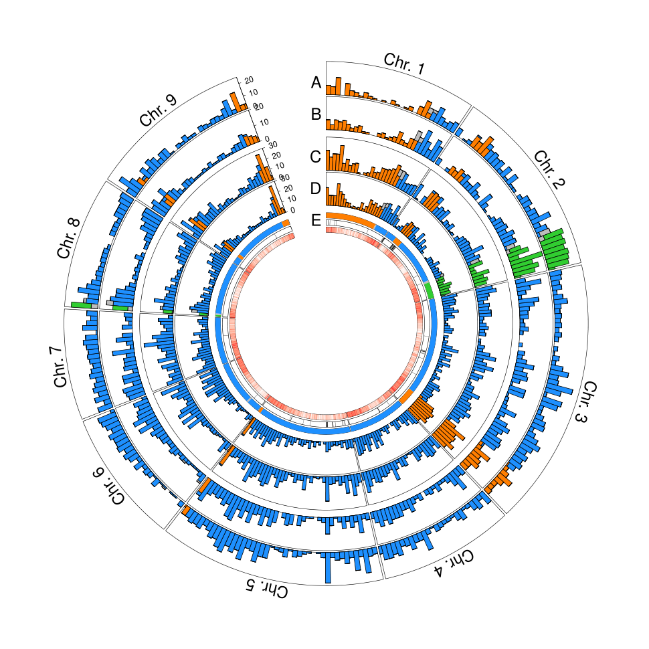


**Figure S4: Distribution of DEGs between sweet orange and its progenitor species.** The number of DEGs along the genome is represented in four different comparisons. Bar height represents the number of DEGs in a given windows, with color representing the admixture pattern of these genes (blue: admixed, orange: non-admixed mandarin, green: non-admixed pummelo). Bars with two colors represent genomic windows spanning two different admixed regions and include genes with both ancestries. A: Flavedo of sweet orange and wild mandarin. B: Pulp of sweet orange and wild mandarin. C: Flavedo of pummelo and sweet orange. D: Pulp of pummelo and sweet orange. E: admixture patterns of the sweet orange genome and distribution of runs of homozygosity (in black) and genic abundance (in red).


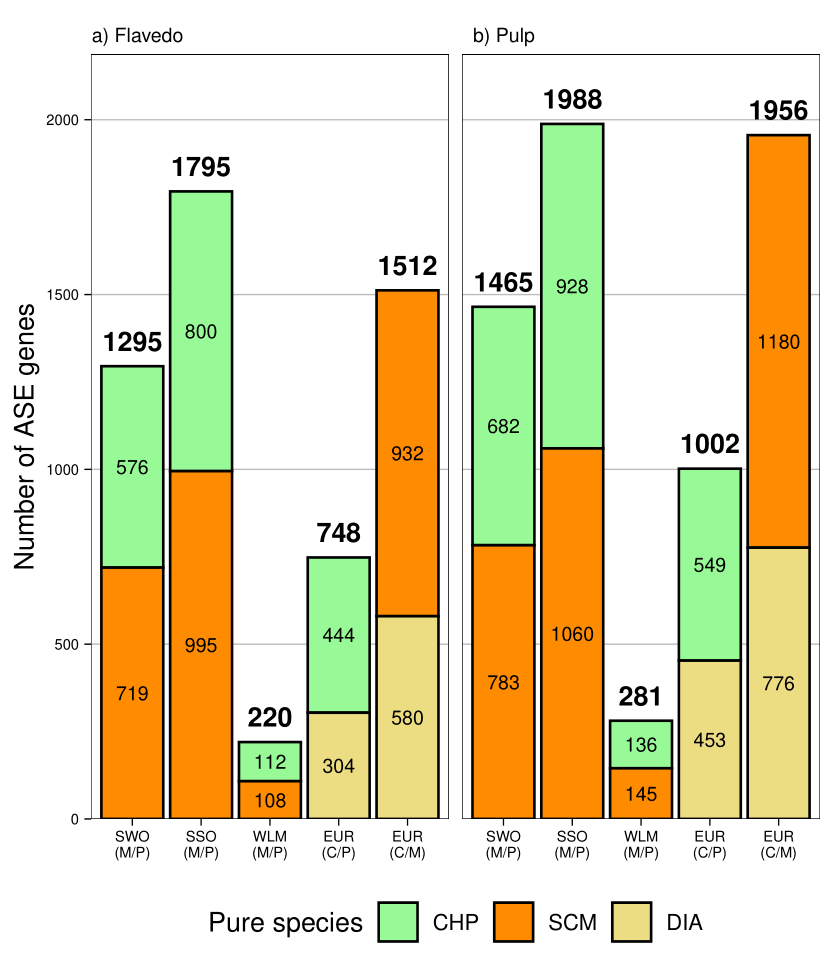


**Figure S5: Allele-specific expressed genes across tissues and cultivars.** Total ASE genes preferentially expressing the citron, pummelo and mandarin in each sample, shown in green, orange and yellow, respectively. For each sample, regions with different ancestry were considered independently: M/P corresponds to mandarin/pummelo regions, C/P to citron/pummelo regions and C/M to citron/mandarin regions. The total number of ASE genes is shown on top of each bar. Flavedo (a) and pulp (b) samples are shown independently. CHP: *C. maxima*, DIA: *C. medica*, EUR: *C. limon*, SCM: *C. reticulata*, SSO: *C. aurantium*, SWO: *C. sinensis*, WLM: *C. deliciosa*.


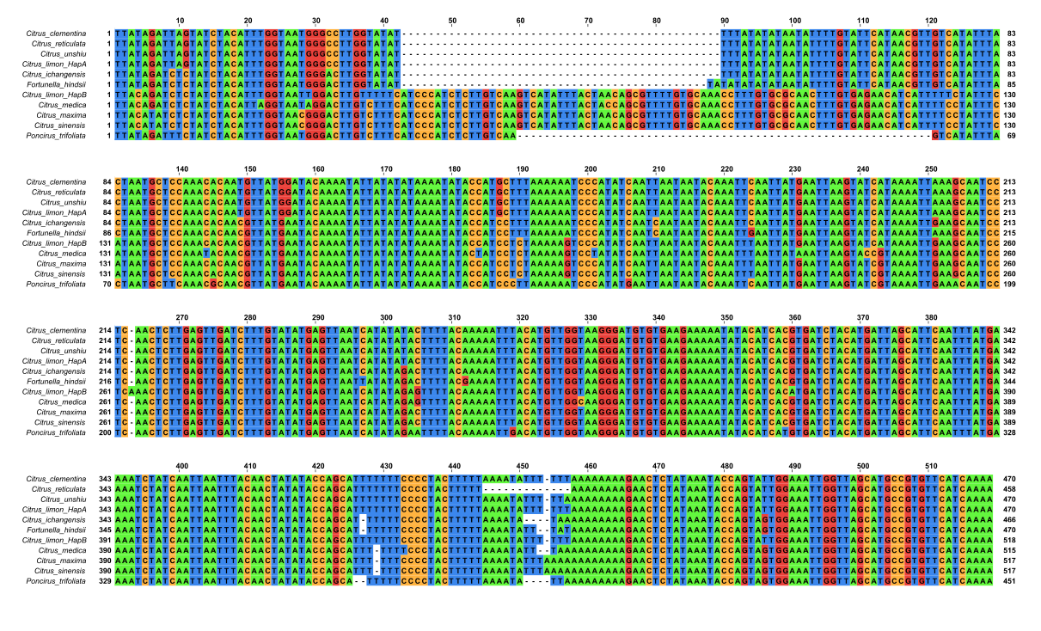


**Figure S6: CHSm promoter sequence.** Nucleotide sequence of 500bp upstream from the gene start in 11 publicly available assembled genomes.
